# LEIGH SYNDROME: A CASE REPORT WITH A MITOCHONDRIAL DNA MUTATION

**DOI:** 10.1590/1984-0462/;2018;36;4;00003

**Published:** 2018

**Authors:** Tânia Lopes, Margarida Coelho, Diana Bordalo, António Bandeira, Anabela Bandeira, Laura Vilarinho, Paula Fonseca, Sónia Carvalho, Cecília Martins, José Gonçalves Oliveira

**Affiliations:** a Centro Hospitalar do Médio Ave, Nova de Famalicão, Portugal.; b Centro Hospitalar do Porto, Porto, Portugal.; c Instituto Nacional de Saúde Doutor Ricardo Jorge, Porto, Portugal.

**Keywords:** ATPase6, Leigh syndrome, Mitochondrial cytopathy, Infant, ATPase6, Citopatia mitocondrial, Síndrome de Leigh, Lactente

## Abstract

**Objective:**

Leigh syndrome is a neurodegenerative disorder with an incidence of 1:40,000 live births. It presents wide clinical, biochemical, and genetic heterogeneity, but with homogenous neuropatoradiological alterations. There is no specific treatment, and the prognosis is reserved. This case report aimed familiarize health professionals with the disease.

**Case Description:**

A 16-month-hold girl who was followed in outpatient clinic due to axial hypotonia and delayed psychomotor development. Karyotype, auditory evoked potentials and ophthalmologic evaluation were normal. Evidence of hyperlactacidemia and hypocitrullinemia was detected in the patient. After performing brain magnetic resonance under anesthesia, hypotonia got worse, and the patient was hospitalized after an episode of cyanosis and apnea. The electroencephalogram showed no epileptiform activity. Neuroimaging revealed bilateral lenticular hyperintensity, especially in the putamen and in the left globus pallidus regions. Molecular analysis revealed an 8993T>G (MT-ATP6) mutation in the mitochondrial DNA.

**Comments:**

Between 10 and 30% of individuals with Leigh syndrome have mitochondrial DNA mutations. The decompensation after anesthetic intercurrences is typically associated with neurological deterioration and, in this case, increased the diagnosis suspicion. It is important to alert for similar cases and to reduce invasive diagnostic tests if the diagnosis is suspected.

## RESUMO

**Objetivo:**

A síndrome de Leigh é uma doença neurodegenerativa com incidência de 1:40.000 nados-vivos. Apresenta ampla heterogeneidade clínica, bioquímica e genética, mas com alterações neuropatorradiológicas homogêneas. Não existe tratamento específico, e o prognóstico é reservado. O objetivo deste estudo foi familiarizar os profissionais de saúde com a doença.

**Descrição do caso:**

Menina de 16 meses, com hipotonia axial e atraso do desenvolvimento psicomotor. Dos exames realizados: cariótipo, potenciais auditivos evocados e avaliação oftalmológica normais; presença de hiperlactacidemia e hipocitrulinemia. Após a realização de ressonância magnética cerebral sob anestesia, observou-se agravamento da hipotonia com necessidade de internação por episódios de cianose/apneia. O eletroencefalograma não mostrou atividade epileptiforme. A neuroimagem revelou hipersinal lenticular bilateral com lesão do putâmen e do globo pálido esquerdo. Encontrou-se a mutação 8993T>G (MT-ATP6) no DNA mitocondrial.

**Comentários:**

De 10 a 30% dos doentes com síndrome de Leigh apresentam mutações do DNA mitocondrial. A descompensação com agravamento neurológico após intervenção anestésica está descrita e, nesse caso, apoiou o diagnóstico. Importante alertar para casos semelhantes, com diminuição de exames invasivos para diagnóstico.

## INTRODUCTION

Leigh syndrome (LS) is a hereditary neurometabolic disease, also known as subacute necrotizing encephalomyelopathy, and was described by the British neuropathologist and psychiatrist Denis Leigh in 1951. It is a neurodegenerative disease with variable symptoms that occurs due to a mitochondrial dysfunction caused by a hereditary genetic defect, associated with bilateral central nervous system lesions. Although it is a rare disease, with an approximate incidence of 1:40,000 live births, it is the most frequent mitochondrial disease in the first year of life.^1^ ^,^ ^2^ ^,^ ^3^ ^,^ ^4^ ^,^ ^5^ ^,^ ^6^ ^,^ ^7^

The heterogeneous functional nature of the mitochondria is responsible for the broad spectrum of clinical manifestations that characterize LS. A dysfunction is present in a restricted but vital area of mitochondrial metabolism, oxidative phosphorylation, in which most cellular adenosine triphosphate (ATP) is produced. Thus, any organ can be affected, but tissues with higher oxygen needs, such as the skeletal muscle, the heart and the nervous system, are usually the most affected.^8^ ^,^ ^9^

The association of neurological symptoms and signs, which can not be explained in terms of the anatomical topography of lesions or because they preferentially reach specific systems, may evoke this diagnosis, and there are no specific clinical signs of mitochondrial cytopathy.^8^ ^,^ ^9^ Although most of the symptomatology is neurologic, some patients may present non-neurological manifestations or even multisystem involvement.^2^ ^,^ ^4^ ^,^ ^10^ Neurological manifestations may include delayed psychomotor development, muscle weakness, hypotonia, dystonia, spasticity, epilepsy, ataxia, intentional tremor, nystagmus, ophthalmoparasia, optic atrophy, dysphagia, respiratory impairment, deafness, paralysis of peripheral cranial nerves, polyneuropathy and myopathy. The most frequent non-neurological manifestations include dysmorphic and endocrine (short stature, hypertrichosis, diabetes), cardiac (dilated or hypertrophic cardiomyopathy) or gastrointestinal abnormalities (diarrhea, vomiting).^2^ ^,^ ^4^ ^,^ ^10^

In LS, aside from the wide clinical heterogeneity, there are also genetic and biochemical variabilities, which contrast with the neuropatoradiological homogeneity.^1^ ^,^ ^2^ ^,^ ^3^ ^,^ ^4^ ^,^ ^5^ The genetic etiology is confirmed in about 50% of cases, with more than 60 mutations identified in nuclear or mitochondrial DNA, the latter being responsible for about 10 to 30% of the cases.^1^ ^,^ ^2^ ^,^ ^3^ ^,^ ^6^ ^,^ ^7^ ^,^ ^11^ One of the most frequently mutated mitochondrial genes is the ATPase6 (MT ATP6) gene, which encodes a subunit of complex V of the respiratory chain, with the most frequently described mutation being the 8993T>G transversion.^2^ ^,^ ^4^ ^,^ ^5^ ^,^ ^9^ ^,^ ^12^ Although hypocitrulinaemia (≤12 µmol/L) is an occasional finding in mitochondrial diseases, it has been specifically associated with the 8993T>G mutation; however, its prevalence is unknown.^6^ ^,^ ^12^ Other biochemical markers that are suggestive of LS are high plasma lactate levels (by glucose overload) and increased lactate/pyruvate ratio; however, their absence does not exclude the diagnosis.^1^ ^,^ ^6^

Faced with clinical and laboratorial suspicion of LS, cranioencephalic magnetic resonance imaging (MRI) should be performed. The most common findings in T2-weighted imaging are focal, bilateral, and symmetric hyperintensities typically located in the basal ganglia (especially the putamen) and/or in the brainstem. Other frequently involved areas are the thalamus, substantia nigra, red nucleus, brainstem, cerebellum, cerebral white matter, or spinal cord.^2^ ^,^ ^6^ ^,^ ^7^ ^,^ ^13^ These lesions, evident both in the brain imaging and i then anatomopathological studies, are attributed to ATP depletion, with consequent lactoacidosis, vascular congestion, hypoxia and, finally, necrosis. The preferential involvement of the subcortical regions is attributed to the greater vulnerability to lactoacidose, which seems to be secondary to its vascular support, the penetrating arterioles.^2^ ^,^ ^4^ ^,^ ^14^

The prognosis of LS is reserved and there is no specific treatment, and multidisciplinary palliative care should be performed.^1^

## CASE REPORT

Female infant, referred to pediatric consultation at eight months of age due to axial hypotonia and Global Psychomotor Development Retardation (PDR). She did not present any relevant perinatal (somatometry at birth was appropriate to gestational age), personal or family antecedents (no history of consanguinity). The objective examination confirmed hypotonia, but showed no other peculiarities, namely, no dysmorphia. An analytical investigation was carried out, which revealed no alterations, namely: CBC, glucose, creatinine, urea, sodium, potassium, chlorine, calcium, phosphorus, creatine kinase (CK), lactate dehydrogenase (LDH), transaminase, alkaline phosphatase (ALP), lipid profile, thyroid function, and venous blood gases. Type II urine/urinary sediment, transfontanelar echography, and karyotype (46, XX in peripheral blood) tests were also performed, which were also normal.

At nine months of age, the patient started physiotherapy thrice a week, having completed six months of treatment. Only mildly improved hypotonia was observed, with maintenance of PDR, which is why CE-MRI was chosen.

At 16 months of age, one day after performing the CE-MRI with anesthesia, the child was admitted to the emergency service (ES) due to a hyporativity episode. There was a parental notion of greater prostration in the hours preceding that episode. Objectively, on admission, there was little reactivity, aggravated axial hypotonia, increased osteotendinous reflexes, and cutaneous pallor. The analytical investigation (which was still within normality) was repeated, a urine drug screening (negative) was performed, and the patient’s hospitalization surveillance was chosen.

On the first day of hospitalization, there was an episode of generalized cyanosis and apnea, with spontaneous recovery followed by somnolence and prostration. An electroencephalogram was performed, which showed a globally altered trajectory, with low amplitude and poor definition of the physiological elements. Treatment with phenytoin was started at 10 mg/kg/day, with no recurrence of the episodes; however, severe axial hypotonia and prostration were maintained, with poor social interaction.

**Figure 1:**
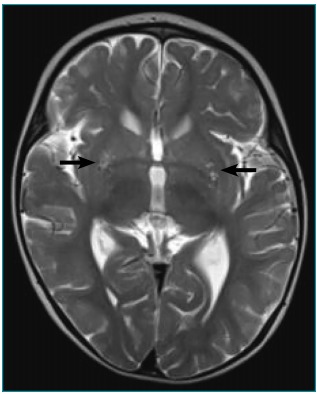
Axial cut of the cranioencephalic magnetic resonance (T2) of the patient with Leigh syndrome and 8993T>G mutation. The arrows show the bilateral lenticular hypersignal.

In the T2-weighted CE-MRI images, bilateral and lenticular hypersignal was observed, expressed in the putamen, and there was doubt regarding the left pallid globe, which suggested the diagnosis of metabolic disease, especially in the absence of intercurrences during gestation and peripartum (Figures 1 e 2). From the metabolic investigation performed, hypocitrulinaemia at 5 µmol/L (normal levels are 15 to 30 µmol/L) and hyperlactacidemia at 3.0 mmol/L (normal levels are 0.5 to 2.2 mmol/L). The patient presented normal serum pyruvate, ammonia, and organic acid chromatography. Evoked potentials of the brainstem, electrocardiogram, and echocardiogram were also performed, which did not show any alterations. She was evaluated by an ophthalmologist, and the examination was within normality.

**Figure 2:**
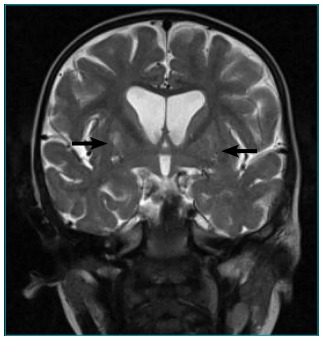
Coronal cut of the cranioencephalic magnetic resonance (T2 weighting) of the patient with Leigh syndrome and 8993T>G mutation. The arrows show the bilateral lenticular hypersignal.

Considering the clinical, laboratory, and imaging data and the suspicion of LS, a panel of mitochondrial genes was carried out, with the identification of the 8993T>G mitochondrial DNA mutation (MT ATP6; heteroplasmy greater than 90%), thus confirming the diagnostic hypothesis. She was referred to consultation at the Reference Center for Hereditary Metabolism Diseases, and was given vitamin supplementation with thiamine, riboflavin, and coenzyme Q10. Parents were informed about the risk of sedation or anesthesia. The maternal family investigation was positive for the same mutation, with 75% heteroplasmy.

During follow-up, the child developed ataxia, dystonia, and epilepsy with a myoclonic component. There was therapeutic substitution for phenobarbital and levetiracetam, with good response. She underwent physical therapy and began occupational therapy.

## DISCUSSION

Although LS is a mitochondrial disease, it is not characterized by the specific clinical signs of this group of diseases, which makes its diagnosis a medical challenge. The wide clinical, laboratorial, and genetic variability should be known, in order to enable earlier diagnosis and, thus, to improve the quality of life.^1^ ^,^ ^2^ ^,^ ^3^ ^,^ ^4^ ^,^ ^5^ ^,^ ^6^ ^,^ ^8^ ^,^ ^9^ ^,^ ^10^ ^,^ ^11^

This is the report of an LS case with onset of symptoms under two years of age, that is, the most frequent variant of the disease: the infant form.^1^ ^,^ ^3^ ^,^ ^5^ ^,^ ^6^ ^,^ ^10^ ^,^ ^11^ Usually, LS manifests itself with a progressive decline of central nervous system function.^2^ ^,^ ^10^ In the case presented, the initial symptoms were PDR and axial hypotonia, and the subacute presentation of the disease was considered.^1^ ^,^ ^3^ ^,^ ^5^ ^,^ ^6^ ^,^ ^10^ ^,^ ^11^

During the first months of follow-up of the patient and once she presented a normal analytical and karyotype investigation, as well as normal transfontanelar ultrasound, an expectant follow-up with physiotherapy was chosen. However, as there was no significant improvement, the study of central hypotonia was started with the application of CE-MRI.

The initial diagnostic suspicion of LS appeared one day after the CE-MRI, in which the patient was submitted to anesthesia with consequent neurological aggravation. In this case, this aggravation arose from metabolic stress induced by the anesthetic procedure, but it may be induced by infections, vaccination, or periods of fasting, which is usual in patients with this entity.^1^ ^,^ ^3^ ^,^ ^5^ ^,^ ^6^ ^,^ ^10^ ^,^ ^11^

The neuroimaging alterations found, along with hyperlactacidemia and hypocitrulinaemia, reinforced the diagnostic hypothesis. For this reason, the genetic investigation focused on the mitochondrial panel of genes, with the 8993T>G mutation. Although more studies are needed, this mutation should be considered early in the diagnostic evaluation of childhood mitochondrial diseases with hypocitrulinaemia, which minimizes the need for invasive procedures, such as muscle biopsies, associated with a small but not negligible risk of complications.^6^ ^,^ ^12^ It is known that the supply of citrulline through diet is minimal, and enterocytes are the main site of its synthesis (via proline ornithine citrulline, which requires the carbamylphosphate I-ATP-dependent reaction). Thus, in LS, hypocitrinemia is thought to be related to altered intestinal biosynthesis of citrulline, secondary to the lack of ATP in enterocytes.^12^

At the time of diagnosis, the ophthalmologic involvement of the cardiac muscle (normal electrocardiogram and echocardiogram), musculoskeletal involvement (normal CK) and brainstem involvement (normal evoked auditory potentials) were excluded. During the follow-up, the development of ataxia, dystonia, and epilepsy were observed, which are described in LS patients.^2^ ^,^ ^10^ However, the screening of other manifestations with which LS can occur, neurological or otherwise, remains important during the course of follow-up, as these manifestations may appear later in evolution.^2^ ^,^ ^4^ ^,^ ^10^

Neuropathy, ataxia and retinitis pigmentosa syndrome (NARP), also associated with the 8993T>G mutation in the MT-ATP6 mitochondrial gene, usually occurs in young adults, as a combination of salt-and-pepper retinopathy, muscle weakness, ataxia, and sensory neuropathy. This mutation occurs in 8 to 10% of LS cases, representing a more severe phenotypic presentation of NARP.^15^

Although there is no curative therapy for LS, multidisciplinary palliative care is essential. The use of some substances has been proposed for the treatment of LS, such as coenzyme Q10, carnitine, lipoic acid, biotin, riboflavin and others; however, there is no clear evidence of their effectiveness.^1^ ^,^ ^6^ ^,^ ^14^ In the case presented, supplementation with thiamine, riboflavin, and coenzyme Q10 was performed, without impediment of the progressive aggravation that is characteristic of LS.^1^ The average survival of patients with LS in childhood is five years, and the current age of the patient described here is three years.^2^

Prenatal diagnosis (PND) is rarely performed in mitochondrial cytopathies with mitochondrial DNA mutation, due to the low reliability of the procedure, since the percentage of heteroplasm varies throughout gestation.^2^ ^,^ ^6^ However, exceptionally, the PND for this mutation may be made available due to the high percentage presented in all organs in the affected cases.^16^ In this way, and considering that the mother has the same mutation, the genetic counseling of this family is of extreme importance.

In conclusion, the authors want to alert to the importance of early recognition of LS, allowing maximization of the quality of life of these patients and their families.
